
JOURNAL INFORMATION
==============================
NLM Title Abbreviation: ScientificWorldJournal
Journal ID: ScientificWorldJournal
NLM Journal ID: 101131163
Journal ID: TSWJ

The Scientific World Journal

ISSN: 2356-6140
EISSN: 1537-744X
Publisher: Wiley

ARTICLE INFORMATION
==============================
PMCID: PMC6009445
PMID: 29973825
DOI: 10.1100/tsw.2002.50
Article ID: 528250
Article version: 1
Subjects: Short Report

Crystal Structure of Rhodopsin: Implication for Vision and Beyond. Mechanisms of Acti

Palczewski Krzysztof *
Departments of Ophthalmology, Pharmacology, Chemistry, University of Washington, Seattle, WA 98195, USA
*Krzysztof Palczewski: palczews@u.washington.edu
Publication date: 2002
Electronic publication date: 2002 Jan 5
Volume: 2
First page: 106
Last page: 107
Copyright: Copyright © 2002 Krzysztof Palczewski.
Copyright year: 2002
License: This is an open access article distributed under the Creative Commons Attribution License, which permits unrestricted use, distribution, and reproduction in any medium, provided the original work is properly cited.
License URL: https://creativecommons.org/licenses/by/3.0/

Funding: National Institutes of Health EY09339 Department of Ophthalmology at the University of Washington Foundation Fighting Blindness Ruth and Milton Steinbach Fund E. K. Bishop Foundation

==============================
Advances in Gene Technology: The Genome and Beyond –
Structural Biology for Medicine (Proceedings of the 2002 Miami
*Nature Biotechnology* Winter Symposium)
*TheScientificWorld* 2002, 2(S2), 106–107
ISSN 1532-2246; DOI 10.1100/tsw.2002.50

### CRYSTAL STRUCTURE OF RHODOPSIN: IMPLICATION FOR VISION AND BEYOND. MECHANISMS OF ACTIVATION

Krzysztof Palczewski
Departments of Ophthalmology, Pharmacology, Chemistry, University of Washington, Seattle, WA 98195
palczews@u.washington.edu

**INTRODUCTION.** The G-protein-coupled receptor (GPCRs) superfamily comprises one of the most diverse groups of signaling molecules involved in numerous physiological processes. Binding of specific ligands to the extracellular or transmembrane domains causes conformational changes that act as a switch to relay the signal to heterotrimeric guanine nucleotide-binding proteins (G-proteins), which in turn evoke subsequent intracellular responses. More than 400 nonsensory GPCRs are also of great interest in medicine, because a vast number of therapeutic agents (>50%) are directed toward these receptors. The largest subfamily, constituting ~90% of all G-protein-coupled receptors (GPCRs), includes rhodopsins, cone pigments, and adrenergic and other receptors. Rhodopsin is involved in photon absorption in retinal rod cells and activation of transducin, a photoreceptor specific G-protein. Understanding of the conformational transformation of inactive GPCR into an activated form capable of interacting with G-protein and signal transduction is a key problem in elucidating molecular steps of cell-surface receptor signaling. The resolution of the X-ray structure of the first GPCR, rhodopsin, provides new insights on the molecular mechanism of activation[1-5].

**METHODS**. Chemical synthesis of retinoids, G-protein activation assay, and other experimental procedures were published recently[6]. Coordinations for bovine rhodopsin were taken from the Protein Data Bank (1F88). Addition of hydrogen atoms and all optimizations were done in Insight II (Insight II release 98.0 [1998], Molecular Simulations Inc., San Diego, CA). The protonation state of amino acid side chains was obtained using standard p$K_a$ values and a pH of 7.0. For molecular dynamics runs with FDiscover (Discover version 2.98, Molecular Simulations Inc.). Retinal and its four locked analogs together with $Lys^{296}$ were optimized with the rest of the protein fixed. The same procedure was applied to residues near (4.5 Å) the movable part of rhodopsin structure. Then the complete protein structure was optimized without constraints[6].

**RESULTS.** Rhodopsin regenerated with a ring-constrained 11-*cis*-retinal analog undergoes photoisomerization; however, it remains marginally active because isomerization occurs without the chromophore-induced conformational change of the opsin moiety. Modeling the locked chromophore analogs in the active site of rhodopsin suggests that the β-ionone ring rotates but is largely confined within the binding site of the natural 11-*cis*-retinal chromophore (Fig. 1).

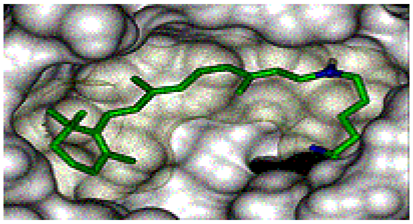

**FIGURE 1**. The structure of 11-*cis*-retinal and in the binding pocket of bovine opsin. The starting model was the PDB entry 1F88. Addition of hydrogen atoms and all optimizations were done in Insight II (InsightII release 98.0 [1998], Molecular Simulations Inc, San Diego, CA). The protonation state of amino acid side chains was obtained using standard $pK_a$ values and a pH of 7.0. For molecular dynamics runs with FDiscover (Discover ver. 2.98, Molecular Simulations Inc., San Diego, CA).(The figure was generated by Dr. S. Filipek).

**DICUSSION.** These results suggest that the native chromophore *cis-trans* isomerization is merely a mechanism for repositioning of the β-ionone ring, which ultimately leads to helix movements and determines receptor activation. Movement of helix VI away from the bundle of other helices could open up the binding site for transducin and activation of this G-protein. Biophysical and biochemical studies of rhodopsin activation carried out by many laboratories, and the recent crystal structure determination of bovine rhodopsin, have improved our understanding on the mechanism of vertebrate rhodopsin activation. In addition, studies on rhodopsin provide a structural and functional template for other members of the GPCR family.

**ACKNOWLEDGEMENTS.** I would like to thank all my collaborators for their contributions to this project. This work was supported by National Institutes of Health Grant EY09339, a grant from Research to Prevent Blindness, Inc. to the Department of Ophthalmology at the University of Washington, and grants from the Foundation Fighting Blindness, Inc., the Ruth and Milton Steinbach Fund, and the E. K. Bishop Foundation.

**REFERENCES**

1. Palczewski, K., Kumasaka, T., Hori, T., Behnke, C.A., Motoshima, H., Fox, B.A., Le Trong, I., Teller, D.C., Okada, T., Stenkamp, R.E., Yamamoto, M., and Miyano, M. (2000) Science 289, 739–745.
2. Okada, T., Le Trong, I., Fox, B.A., Behnke, C.A., Stenkamp, R.E., and Palczewski, K. (2000) J. Struct. Biol. 130, 73–80.
3. Okada, T. and Palczewski, K. (2001) Curr. Opin. Struct. Biol. 11, 420–426.
4. Okada, T., Ernst, O.P., Palczewski, K., and Hofmann, K.P. (2001) Trends Biochem. Sci. 26, 318–324.
5. Ballesteros, J. and Palczewski, K. (2001) Curr. Opin. Drug Discovery Dev. 4, 561–574.
6. Jang, G.F., Kuksa, V., Filipek, S., Bartl, F., Ritter, E., Gelb, M.H., Hofmann, K.P., and Palczewski, K. (2001) J. Biol. Chem. 276, 26148–26153.
